# The efficacy assessment of emamectin benzoate using micro injection system to control red palm weevil

**DOI:** 10.1016/j.heliyon.2019.e01833

**Published:** 2019-06-23

**Authors:** Mona Mohamad Mashal, Basil Faisal Obeidat

**Affiliations:** National Agricultural Research Centre (NARC), P.O Box: 639, Baq'a, 19381, Jordan

**Keywords:** Agriculture

## Abstract

Red palm weevil is the most injurious pest on dates globally. The purpose of this field trial was to evaluate the preventative & curative effect of the micro emulsifier insecticide Emamectin Benzoate in two formulations: Revive® 4 % and ReviveII® 9.5 % against the red palm weevil for one year. A completely randomized block design was applied on 36 mid to high infested trees with 4%,9.5% and the control. One single direct micro-injection was applied at the base of the trunk using Syngenta TMI 4.1 device, under low pressure of 2 bar. Biweekly monitoring for Red palm weevil external symptoms of treated trees. Treated Trees were cut and dissected after: 3, 6, & 12 months from injection date collecting all RPW individuals from the out side and the inside of the tree trunk, it was found that RPW mean mortality% cause by Revive was 88.1 and 98.8for ReviveII®9.5%. descriptive symptom data and RPW mortality% inside the trunks showed that trees injected by Revive®4% and ReviveII®9.5% were cured 100% from RPW for one year by killing renewable infestation. LOQ of Emamectin benzoate were quantified in fruit and compared with MRL level after 60 and 100 days. Results indicated that no residues of ReviveII® in fruit samples after 60days.

## 1 Introduction

The Red Palm Weevil (RPW) *Rhynchophorus ferrugineus* Olivier (Coleoptera: Curculionidae) is known as the cancer of palm trees. It is the most dangerous and destructive pest to ornamental palm trees and date palm trees [1]. It affects more than 40 species of palm trees across 50 countries. Hundreds of thousands of infested palm trees die because of the effect of RPW [2, 3, 4, 5] with continuous losses every year. The RPW is becoming a true concern and causes significant losses financially, environmentally and economically. In the Hashemite Kingdom of Jordan, 7000 Palm Trees have been lost because of the RPW, and the number is constantly growing [4, 5, 6].

The RPW spends the early and middle stages of its lifecycle inside the trunk of palm trees which results in significant damage to the inner tissues of the tree trunks, and eventually leads to the death of the tree. It is difficult to detect infection and carry diagnosis at the early stages of infestation. The larvae are responsible for damaging the tree and they eventually cause the death of the palm tree, while the adult is responsible for renewing the infestation. The adults fly and mate resulting in larvae hatching and spreading among the trees, by selecting a new host and depositing thousands of eggs in the tree trunks [7, 8]. These eggs hatch within 2–5 days resulting in legless grubs that bore into the interior of the palms, feeding on the soft tissues and discarding all fibrous material. The larval stage varies from 1 to 3 months and they pupate in cylindrical cocoons made of fibrous strands. At the end of the pupation period which lasts anywhere from 14 to 21 days, adults emerge and fly out the tree searching for a new host and a potential mate, where the process is repeated and damage to a new tree spreads [7, 8] Control strategies for RPW are based on integrated pest management mainly on monitoring by pheromone traps [9], protection by regulations such as agricultural quarantine and orchard sanitation with good cultural practices [10], while the treatment depends on chemical application by spraying, irrigating and injecting lots of insecticides [1].

The use of chemical application is the most dominant effective method used to control the pest. Contact insecticides are applied through spraying methods, while systematic insecticides are applied either though injection in the trunk or through soil irrigation [1, 6, 7]. The insecticide application should be repeated every three weeks to three months depending on the type of insecticide and the method of application [7]. This excessive use of insecticides contaminates the environment [6, 9, 10], as the chances of insecticides particles spreading increases which may lead to a hazard case to the bio surroundings, and affect the safety of the fruit due to high MRL in the fruits of treated trees [7, 9].

Syngenta crop protection developed the tree micro-injection technique using Revive®4% & Revive II® 9.5%, a chemical product containing emamectin benzoate. This compound is the Avermectin class of insecticides, which is composed of a active substance of natural origin. The Emamectin insecticide is produced after a microbial fermentation process of Streptomyces Avermitilis [10, 11].

Moreover, Emamectin kills the insect by disrupting neurotransmitters, causing irreversible paralysis. It is lethal upon ingestion or direct contact [6]. The Emamectin acts as a GABA agonist and binds to the GABA receptor complex [11]: this results in channel opening, hence mimicking the action of the inhibitory neurotransmitter GABA [11, 12]. Revive & Revive II are specifically designed for Tree Micro Injection allowing low pressure application of tiny volumes of the insecticide to be applied in the two formulas (Revive® 4% & Revive II® 9.5 %) [12] as a component of IPM to kill the RPW by using fast injection method with non-diluted homogenous insecticide [13]. The injection is done by using a specific injector, developed by Syngenta (TMI) [13, 14]. This Treatment is inserted directly into the palm trees with null damage were observed on treated palms, no exposure to the public, or environment [14].

Syngenta recommends a single micro-injection application for effective and reliable control of RPW for up to 12 months. Revive has passed rigorous testing and risk assessments for safe environmental impact, it is also proved to be non-harmful for pollinator insects and pets as well as palm tree health [6].

Furthermore, the injection of a novel Emamectin Benzoate 40 g L (-1) liquid formulation, {Shot Wan Liquid Formulation} exerted a preventative effect against the Pine Wilt disease, caused by the pine wood nematode Bursaphelenchus Xylophilus [14, 15], and this effect lasted for at least 3 years [14, 15, 16, 17]. Revive has also credited for saving southern Europe's iconic palm trees from the devastating effects of the RPW Rhynchophorus ferrugineus [13, 18].

Moreover, it has been fully approved in France and granted emergency approval to tackle the increasing pest pressure in Spain. The treatment is claimed to provide a fast, efficient and unobtrusive treatment, with minimal disruption or interference to the trees, the public or the environment and just one single targeted treatment can stop RPW activity in the tree for a whole season [19] And also it was used efficiently to control Diamondback moth and other crucifer pests [13].

Controlling RPW results by Revive micro-injection application in Europe encouraged us to evaluate Revive, to control RPW, the most destructive pest on palm trees in Jordan and all Arab region country as well as all palm planted regions in the world [20].

It is necessary to have an effective insecticide with simple application method and having the ability to reach and kill the different stages of the date palm weevil individuals inside the tree and has to be safe for human health and doesn't environmental hazards.

## 2 Methods

The aim of this experiment was the evaluation of the two formulas of micro emulsion formulation of emamectin benzoate (Revive® 4% 40 g/*L emamectin* benzoate& Revive® II 9.5 %95 g/*L emamectin* benzoate) (Syngenta Crop Protection AG, Switzerland) to control red palm weevil individuals inside the treated trees before and after single application using injection (Syngenta TMI 4.1 device), the effect of this single treatment was biweekly externally trees monitored for one year, treated trees was dissected after three, six and twelve months and then it was made a comparison to a non treated trees.

### 2.1 Implementation of the experiment

A site selection was done by choosing Berhi cultivar date palm orchard, which is severely infested with RPW in a highly infested area in Jordan Valley. All trees in the orchard were infested; while 50% of them were dead and cut before and during the experiment. This situation caused a challenge to apply this experiment due to a constant source of infestation, and renewal of continuous infestation affecting the treated trees.

The experiment was carried out by selecting 36 medium to high recognized RPW infested date palm trees of similar age (15 years old) and almost the same width and height of trunk (2 m height) with significant conspicuous symptom like holes, sawdust, leaf yellowing, etc... Noting that the selected trees although infested were still growing and green. The experiment was done for a year, starting on 29^th^ March 2017 and ending on 8^th^ April 2018. The treatments were conducted using a completely randomized design, with three main divisions each containing 12 trees. The first division was treated with Revive (4%), the second division treated with Revive II (9.5%) and the third division as a control without any treatment.

The treated trees, a total of 24, had four holes drilled into each tree, using an electrical drill bit 8 mm in width, in length ranging from 20 – 30 cm but no more than 1/3 the trunks’ diameter at a 10–20° angle downwards into the base of the stem. The holes were drilled around the tree, at the same level, at the base of palm trunk. The undiluted product Revive: 48 ml/palm & Revive II: 24 ml/palm (2 g a. s./palm) was injected into each hole using Syngenta TMI 4.1 device. A total of 48ml of Revive® 4% was used per tree (12 ml per hole), while a total of 24ml per tree was used of Revive II®9.5% (6 ml per hole). After injection the holes were closed with sterilized wooden plugs to protect the tree from contamination and avoid pesticide reflux. The last twelve trees were not injected and were used as a control measure.

The injection was done under low pressure of 2 bar so as not to disturb the tissues of the plant. Due to lack of commitment from the farmer, some of the recovered trees were sold by the farmer prior to experiment ending, a second injection of six new trees was carried out using Revive II on August 2017.

### 2.2 Periodic observation

In order to detect the long term effect (for a year) of the two injected insecticides Revive®4% and Revive®II9.5%, Observations or symptoms monitoring were taken every two weeks for a year, readings were taken for the 36 treated trees of the experiment. Symptoms were recorded like gums, oozing, holes, mold, sawdust and soft or dry tissue, all RPW individuals were collected from the external of the treated tree trunk of the trees and recorded the numbers and if it is alive or dead.

### 2.3 Cutting and dissecting procedure

Cutting and dissecting trees were applied three times; after three, six and twelve months from injection date, Cutting is done for the treated and control trees (three trees for each treatment of total 9 trees at each cutting date). the selected trees were cut at the base of the tree using diesel saw, all leave were removed, all insect individuals were collected from all holes at the trunk outside. After that, the trunk was cut longitudinally for four pieces. Then each pieces was also cut longitudinally and horizontally for small slides then cut to mini parts (5–10cm), all dead and alive RPW individuals were collected and then put inside plastic pots, samples were transferred to the lab, insect individuals were counted and recorded to detect mortality of each treatment.

### 2.4 Curative calculation

Curative effects was measured by collecting all dead, lived and all insect parts of red palm weevil individuals from each treated and control dissected trees (didn't treated), and then counting mortality % using Sun-Shepard's formula [14, 15]

$$\text{Change}\;\text{\%}\;\text{in}\;\text{control}\;\text{plot}\;\text{population}=\left(\frac{\text{Population}\;\text{in}\;\text{control}\;\text{plot}\;\text{after}\;\text{treatment}-\text{Population}\;\text{in}\;\text{control}\;\text{plot}\;\text{before}\;\text{treatment}}{\text{Population}\;\text{in}\;\text{control}\;\text{plot}\;\text{before}\;\text{treatment}}\right)*100$$

### 2.5 Preventative calculation

Preventive calculations [14, 15] were laid on the mortality% that the treated become cure when mortality became high or when the insect individuals were disappeared from the tree and have zero individual as we have got in the most of the dissected treated trees.

### 2.6 Statistical analysis

Statistical Analysis Means percentages of RPW individuals mortality at the three cutting and dissecting dates under randomized complete block design, were subjected to one-way analysis of variance (ANOVA, The significance level was set at 0.05, and the means were separated by LSD.05, discriptive histograms where done to describe the effect of Revive®4% and ReviveII®9.5% on insecticide mortality at the trunk surface and inside, All statistical analyses were performed using the Mstatc-6.1tatistical software.

### 2.7 Emamectin benzoate residues

In order to detect Emamectin benzoate residues of the two formulas; Revive®4% and ReviveII®9.5% inside treated fruits, Level of Quantification (LOQ) was detected and compared with Maximum Residue Limit (MRL) using codex aliment Arius of FAO and EFSA for the result evaluation [3], LOQ is the lowest concentration of the tested samples that can be determined with acceptable precision and accuracy under the stated conditions of test) [13], while the MRL (is the highest level of a pesticide residue that is legally tolerated in or on food or feed when pesticides are applied correctly [20]. The fruit samples were taken after 60 and 100 days of injection date (at fruit cell expansion developing stage named khalal stage which is in the highest fruit activity). Half kilogram fruits were collected randomly from every treated tree in the same treatment ( Revive®4% and ReviveII ®9.5%. two samples of one kilogram were taken from gathered fruits of each treatment sample and stored directly in nylon bags then placed in a refrigerator, The samples were then transferred directly to the local certified laboratory to be analyzed, while the another sample were kept into a refrigerated container and transferred directly To the airport for analysis in the Syngenta Crop Protection AGA application Technology lab, samples were analyzed and determined the insecticide consentration in the fruitsLOQ and then comparing with MRL, the analysis was done using SOP based on BS EN 15662–2008, using LC MSMS device [19, 21].

## 3 Results and discussion

Monitoring the development of RPW external symptoms on treated trees during one year period Tables 1, 2 and 3 represent the development of the external symptoms on the treated trees for one year after the injection of Single Micro Injection of Emamectin, while Table 4 represent the developing of the external symptoms on the control (untreated infested trees), infestation tracking tables have been included here to detect The behavior of the RPW infestation under the treatment of Micro Injection of Emamectin Benzoate in every treated trees, taking into account the specific conditions of each tree. However, all selected trees have mid to high infestation had these following symptoms on the out side of the trunk as holes, oozing, sawdust, wet fermented tissue, yellow of some fronds and the presence of RPW individuals on the outside the trunks, then randomly twenty four trees were injected equally with Revive and Revive II, results in Table 1 indicated that all twelve trees of treatment one (Revive) showed cure from the infestation with continuous significant improvement with time, that all external symptoms disappeared although new RPW infestation attacked the treated trees by lying eggs but the larvae couldn't survive after feeding on the trunk tissues (got the LD50) then stopped feeding & killed which lead to dry ooze & tunnels such as in tree:1,7,8 and 11. Results of the first evaluation (cutting and dissecting) after three months as shown in Table 5 indicated that the collected RPW individuals from outside the three tree trunks whether before or after dissecting revealed excellent efficacy of Revive but the reading of data inside the trunks revealed statistically acceptable control which reached to 85.8% of cure, this result in decreasing the insecticide efficiency because one of the tested trees contained many lived larva inside a very big cavity in the core of the tree that the insecticide solution disabled translocated through the bundle tissue from the injection point at the base of the tree to upward these symptoms were unseen and this is unfortunately couldn't be detected that caused misleading and failed in control this situation could be solved by making injection before and after the infestation level. On the other hand, results of the evaluation of the micro injection of Revive of both second evaluation after six months as the last evaluation after twelve months as in Table 5 revealed that Revive4% succeeded in reaching 100% trees cure, this means that it did not found lived larva out site and inside the trees although some RPW individual were collected at 5cm depth that will rapidly dead.

**Table 1:** Monthly monitoring of RPW external and internal symptoms on the injected tree (Revive®4%).

Date One Year	trees Injected with Revive											
	1	2	3	4	5	6	7	8	9	10	11	12
March/017	e	e	e	e	e	e	e	e	e	e	e	e
April	e	a	a	a	a	a	a	a	a	a	b	a
May	b	a	a	a	a	a	a	a	a	a	b	a
June-3month	b	a	d	a	a	a	a	b		a	a	d
July	b	a		a	a	a	b	a	b	a	a	
August	a	a		a	a	a	b	a	b	a	a	
Sept	a	a		a	a	a	a	a	a	a	a	
Sept	a	a		a	a	a	a	a	a	a	a	
Oct	a	a		a	a	a	b	a	a	a	a	
Oct-After 6month	c	d		a	a	a	d	a	a	a	a	
Nov				a	a	a		a	a	a	a	
Dec				a	a	a		a	a	a	a	
Jan/2018				a	a	a		a	a	a	a	
Feb				a	a	a		a	a	a	a	
March				a	a	a		a	a	a	a	
April-after 12month				a	a	a		a	a	d	d	

a-No Infestation Healthy Growth 100% Cure	b-New Infestation Dried Excellent Growth	c-100% Internal Cure 0% External Cure, Cut	d-100% Internal Cure Cut	e-Med- or high symptom

a No infestation: All out side infestation symptoms by RPW were disappeared and the trunk was healed and the tree appeared healthy.

b New Infestation :The dried excellent growth injected trees (no infestation) was noticed a new out side symptoms on the trunk like gums, oozing, holes, mold, sawdust and soft or dry tissue.

c 100% Internal Cure, 0% External :the tree have outside new infestation and when the tree cut and the trunk was dissected it was revealed that no RPW individuals inside the trees that the larva still not ate the inside poisoned trunk tissues by the pesticide.

d 100% Internal Cure , Cut :the cut tree has no RPW individuals or symptoms at the trunk outside before and after cut when dissecting was applied.

e Med- or high symptom: all selected trees which were chosen to apply the experiment or non respond trees to the treatment of the injection revealed out side symptoms on the trunk like gums, oozing, appeared many holes, trunk cavities ,mold, sawdust and soft or dry tissue(these trees classified as med to high infested trees because the inside trunk cannot be seen.

**Table 2:** Monthly monitoring of RPW external and internal symptoms on the injected tree (Revive II).

Date One Year	Trees injected with Revive II											
	1	2	3	4	5	6	7	8	9	10	11	12
March/017	e	e	e	e	e	e	e	e	e	e	e	e
April	a	a	a	a	a	a	e	b	b	b	b	b
May	a	a	b	a	a	a	e	a	e	a	e	a
June-3month	a	a	d	d	a	a	f	d	f	a	a	a
July	b	a			a	a	f		f	a	a	a
August	b	a			a	a	f		f	a	a	a
Sept	b	b			a	a	f		f	a	a	a
Sept	b	b			a	a	f		f	a	f	f
Oct	b	b			a	a	f		f	a	f	f
Oct-After 6month	b	b			a	a	f		f	a	f	f
Nov	b	b			a	a	f		f	a	f	f
Dec	b	b			a	a	f		f	a	f	f
Jan/2018	b	a			a	a	f		f	a	f	f
Feb	a	a			a	a	f		f	a	f	f
March	a	a			a	b	f		f	b	f	f
April-after 12month	a	a			a	d	f		f	d	f	f

a-No Infestation Healthy Growth 100% Cure	b-New Infestation Dried Excellent Growth	d-100% Internal Cure, Cut	e-Med- or high symptom	f-missed

a No infestation: All out side infestation symptoms by RPW were disappeared and the trunk was healed and the tree appeared healthy.

b New Infestation :The dried excellent growth injected trees(no infestation) was noticed a new out side symptoms on the trunk like gums, oozing, holes, mold, sawdust and soft or dry tissue.

d 100% Internal Cure , Cut :the cut tree has no RPW individuals or symptoms at the trunk outside before and after cut when dissecting was applied.

e Med- or high symptom: all selected trees which were chosen to apply the experiment or non respond trees to the treatment of the injection revealed out side symptoms on the trunk like gums, oozing, appeared many holes, trunk cavities ,mold, sawdust and soft or dry tissue(these trees classified as med to high infested trees because the inside trunk cannot be seen.

**Table 3:** Monthly monitoring of RPW external symptoms on the injected tree (Revive II®9.5%). Compensation of the missing trees in August 2017.

Date	Trees injected with Revive II					
	13	14	15	16	17	18
6/Aug/ Injection	e	e	e	e	e	e
August	b	b	b	b	b	b
Sept	a	a	a	a	a	a
Oct	a	a	a	a	a	a
Nov	a	a	a	a	a	a
Dec	b	a	a	a	a	a
Jan	a	a	a	a	a	a
Feb	a	a	a	a	a	a
March	f	a	a	a	a	a
8April/cut after6 months	f	a	a	d	d	d

a-No Infestation Healthy Growth 100% Cure	b-New Infestation Dried Excellent Growth	d-100% Internal Cure, Cut	e-Med- or high symptom	f-Missed trees

a No infestation: All out side infestation symptoms by RPW were disappeared and the trunk was healed and the tree appeared healthy.

b New Infestation :The dried excellent growth injected trees(no infestation) was noticed a new out side symptoms on the trunk like gums, oozing, holes, mold, sawdust and soft or dry tissue.

d 100% Internal Cure , Cut :the cut tree has no RPW individuals or symptoms at the trunk outside before and after cut when dissecting was applied.

e Med- or high symptom: all selected trees which were chosen to apply the experiment or non respond trees to the treatment of the injection revealed out side symptoms on the trunk like gums, oozing, appeared many holes, trunk cavities ,mold, sawdust and soft or dry tissue(these trees classified as med to high infested trees because the inside trunk cannot be seen.

f missed trees: trees that sold by farmers with out coordination with the experiment team

**Table 4:** Monthly monitoring of RPW external and internal symptom on the trees in control treatment.

Date one year	Control – Non Injected or Treatment											
	1	2	3	4	5	6	7	8	9	10	11	12
March/017	e	e	e	e	e	e	e	e	e	e	e	e
April	e	e	e	e	e	e	e	e	e	e	e	e
May	e	e	e	e	e	e	e	e	e	e	e	e
June-3month	h	h	g	g	e	e	e	e	e	g	e	h
July					e	e	e	e	e		e	
August					e	e	e	e	e		e	
Sept					e	e	e	e	e		e	
Sept					e	e	e	e	e		e	
Oct					e	e	e	e	e		e	
Oct-After 6month					g	e	e	g	g		e	
Nov						e	h				e	
Dec						e					e	
Jan/2018						e					e	
Feb						e					e	
March						e					e	
April-after 12month						g					g	

h-Broken high infested tree	g-Cut: Inside high Infestation	e-Med- or high symptom

h Broken high infested tree :the tree seemed very weak and evacuated from inside with huge cavities inside the trunk ,huge amounts of gums, oozing, holes, mold, sawdust and soft or dry tissue

g Cut: Inside high Infestation: when the tree was cut for dissecting purpose to collect data ,huge numbers of RPW exceeded 50 individuals with huge cavities with disturbed fermented tissues

e Med- or high symptom : all selected trees which were chosen to apply the experiment revealed out side symptoms on the trunk like gums, oozing, appeared many holes, trunk cavities, mold, sawdust and soft or dry tissue(these trees classified as med to high infested trees because the inside trunk cannot be seen.

**Table 5:** Mortality% of RPW individuals collected after cutting and dissecting treated trees in the three evaluation dates.

Treatments	Cutting and dissecting dates (RPW individual mortality%)			
	3 months	6 months	12 months	mean
Revive ®4%	67.8ab	96.6a	100a	88.1
Revive II®9.5%	96.3a	100. a	100a	98.8
Control	3.3c	3.3b	5b	3.9
Anova Within-treatments :Df 24 F 224.82502		The *f*-ratio value is 224.82502. The *p*-value is < .00001. The result is significant at *p* < .05.		

Means within a column not sharing a common letter are significantly different at p < 0.05 using LSD test.

**Fig. 1:**
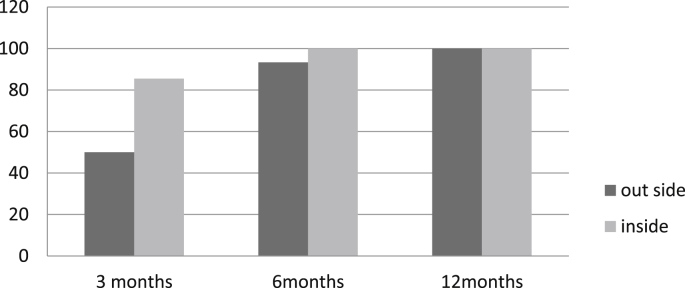
Mortality %of RPW insIde and out side the trunk of the treated trees by Revive®4%for one year.

**Fig. 2:**
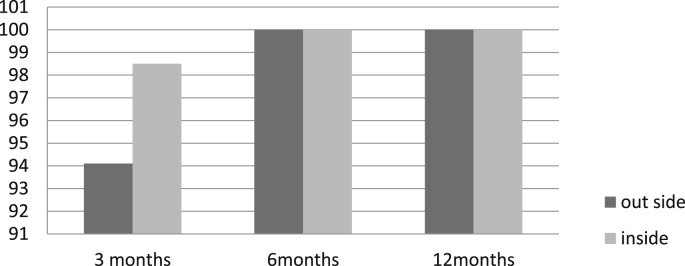
Mortality %of RPW inside and out side the trunk of the treated trees by ReviveII®9.5%for one year.

Tables 2 and 3 represent the monitoring of the efficacy of ReviveII on controlling the infestation of RPW. Four trees were lost, two of them were sold by farmer and the other two broke due to high intensive infestation, which lead to a hollow and empty trunk, these two trees were beyond rescue. This lead to re injection of six trees to avoid missing the treatment. Continuous improvement with continuous renewal of the infestation due to high infested orchard, although most of treated trees became healthy and RPW symptoms disappeared, the tissues of the infested holes and the sawdust dried up. While some trees had renewed infestation, as an occurrence of soft holes that also dried up at the second or third reading, many dead adults were collected while still inside the cocoons. Dead pupae in the first three months were also collected from the infested holes of the outer side of the trunk. This has happened due to gradual poisoning of the larvae which requires a month after treatment. The Emamectin Benzoate is translocated by the tree tissues itself, which means it needs time to spread and reach full concentration at all trunk tissues on all levels. It is crucial to emphasize the degree of damage vs the success of the treatment as the treatment relies heavily on healthy tissue to succeed. On the other hand, the results of the evaluation of the micro injection of Revive and Revive II after three, six and twelve months in Fig. 1 and Fig. 2, shows that the cure reached 98.5% after three months, while after six and twelve months the cure reached 100% meaning that the Revive and Revive II succeeded in control RPW and the cure reached to the highest level. The results indicated that once the insecticide Emamectin Benzoate became inside the tree it became lethal to RPW individuals upon ingestion or direct contact by disrupting neurotransmitters, causing irreversible paralysis.

Also, there is no significant differences after 3, 6 and 12 months as in Table 5 between means of collected RPW individuals after trees cutting and dissecting using LSD. 05 between Revive and ReviveII®9.5%. Although there are some non-significant differences between means of the two treatments after three and six months comparing to highly significant differences between the two treatments versus control treatment using LSD.05.

The control as in Table 4 shows the tracking monitoring of RPW infestation on the trees with no treatment (control) during the year, four trees were broken while the other trees showed very high infestation with no improvement, this is highly predicted in tables of cutting and dissecting results that many of living RPW individuals were collected from dissected trees.

### 3.1 RPW mortality % by Revive®4%, ReviveII®9.5% at cutting and dissecting date

Table 5 shows the RPW means individuals mortality that collected from dissected treated trees which injected by Revive®4%, ReviveII®9.5% comparing with the control at three cutting and dissecting dates, Results of one way ANOVA analysis showed that, there is a significant difference between all means, The *f*-ratio value is 224.82502 so that The *p*-value is <.00001 and the result is significant at *p* < .05. On the other hand, results showed that no significant difference between RPW means mortality (have the same alphabet in the same column) between Revive and ReviveII®9.5% and highly significance with control under the three cutting and dissecting dates using lsd.05 test, although there is a some difference between mortality means under Revive and ReviveII®9.5%, that mortality % were the highest under Revive®4% treatment after 6 months from injection and the lowest after twelve months, while the highest mortality in revive II was after twelve months and the lowest after six months.

In the contrary, Revive®4% and ReviveII®9.5% showed that (Tables 1, 2 and 3) no insect individuals were collected from most inside dissecting trees (exclude one) comparing with the control at three cutting and dissecting dates, the tree that have lived individuals inside its trunk is one the trees that injected with Revive®4% and dissected after three months, it was showed a live larvae inside the trunk at the lower level from injection point and on the upper site of a big evacuated cavity in the trunk which done by highly infestation of RPW that destruct all vascular and became dysfunction that the insecticide couldn't pass through vascular tissue after injection as shown in Fig. 1 (RPW mortality% outside the tree was%50), So this situation statistically highly decreased the mortality% means to 67.8 a shown in Table 5, however, beside the RPW individuals collected from the outside trunk (Fig. 1, Table 5) (comes from renewing the infestation decrease the mortality %, this happened for two trees injected with Revive®4% and dissected after twelve months. In the conclusion, the results in Table 5 detected that Revive with total mortality% mean:88.1) and ReviveII®9.5% with total mortality% mean:98.8 were very effective in killing RPW individuals with statistical priority to ReviveII®9.5%, and the descriptive data showed that the two formulas cured the trees from inside from the RPW infestation after one single injection (Tables 1, 2 and 3) Leaving healed dried tissues with stopping the tunnels inside the trunks although the renewal infestation had done all the time (Fig. 1, 2) but both Revive®4% and ReviveII®9.5% have killed the new invasion larva that tunneled in side the trunk more the 5cm where the two formulas; Revive®4% and ReviveII®9.5%, were affect best (Tables 1, 2 and 3). Further more, the Cure from the renewed infestation were done on all treated trees with low and medium infestation as shown in Fig. 1, while the success in eliminating of the high infestation has done when at least 1/3 of internal (vascular tissue) trunk tissues be stilled there or relatively functioning as what happened in some highly infested trees in the experiment so that the insecticides can translocate through the bundle tissues and reaching the invaded RPW individuals inside the tree. On the other hand, the numbers of collected RPW from the control increased with time to reach the highest infestation peak after six months comparing with the first evaluation date after three months because the intensity of the infestation was increased with time and finally with some reverse in intensity after twelve months due to the high trees deterioration from RPW infestation.

### 3.2 MRL tested in treated fruits

Table 6 represents the insecticide residues inside fruit samples of the treated trees as a reference to LOQ (lower limit of quantification) and compare to the MRL (codex alimentarius (FAO) and European regulation) [18, 19, 22] EFSA afor both Revive®4% and Revive II®9.5% after 60 days and 100 days from injection date, the results indicated that fruits from palms treated with Revive II®9.5% passed the tests of MRL in Jordan lab in both reading dates; 60 and 100 days and in Syngenta lab for samples after 100 days. That no insecticide traces were detected in the tested fruits comparing with LOQ of the Emamectin Benzoate, this result recommended to repeat the test in another experiment in a shorter period less than 60 days, that the safety period for the pesticide may be shorter than 60 days. On the contrary, result of Revive®4% in Jordan lab shows that the concentration of the pesticide was higher than LOQ of the Emamectin benzoate and MRL of and therefore the sample was not identical and failed. However, the concentration of the insecticide in the sample increased after 100 days, this result places a question that the Emamectin benzoate concentration of Revive®4% increased with time, noting that the result of Syngenta lab of Revive after 100 days emphasize this conclusion. It was recommend to repeat this test in another experiment, as the results could have been tampered due to the unscheduled spraying by the farmer.

**Table 6:** MRL Testing in treated fruits using the LC MSMS device.

Insecticide	Date after injection	Concentration LOQ (mg/kg)	**LOQ	*MRL Mg/kg	Pass/Fail Jordan Lab	Pass/Fail Syngenta Lab
Revive®4% 4%Emamectin benzoate 1a	60 days	0.0127	.004	.01	Fail	No sample
	100 days	0.0223			fail	Pass
Revive II®9.5% 9.5% Emamectin benzoate 1b	60 days	------	.01	.01	Pass	No sample
	100 days	-------			Pass	Pass

*MRL (Maximum Residue Limit) ** LOQ (Lower Limit of Quantification that can detected of the pesticide in the sample).

## 4 Conclusion and recommendation

Syngenta Tree-Micro injection using Emamectin Benzoate micro emulsifier in two formulations Revive®4% & ReviveII®9.5% with previlage to ReviveII®9.5% a very promising techniques to control & protect the invasion of RPW the most aggressive insect on date palm trees. It was found that one single injection will be very effective in controlling & protecting from the red palm weevil and keeping palm trees inside free from the RPW injury for one year.

It is a simple sustainable & safe application method which has the ability to reach and kill the different stages of the weevil inside the tree while minimizing the environmental pollution and relatively cost for the farmer (labor, water, equipment & time costs, and repeated insecticide application). Also, it is advisable injection appling coincide with spraying the trunk outside with suitable insecticide or insect repellant to protect any invasion by new RPW individuals that have not been yet reached the inner tissue, or are closer to the outside of the trunk than inside. Taking into consideration the success of this micro injection application should be an integral part of IPM program. It is recommended to inject the treatment in autumn after harvesting date until 60–100 days before harvest time, or in the early season before any rise in RPW population activity inside the orchards. Although no residue that was higher than MRL in the treated fruits after 60 & 100 days of injection date, So that Revive®4% & ReviveII®9.5% injecting in the early season protected and lowering any chances of residual presence in the fruits beside tree controlling protection. It also permits a suitable time for interfering in controlling and suppressing the RPW infestation and distribution before increasing the RPW population and before the natural insect enemies become active with rising temperatures. It is strongly recommended to retest the MRL of Revive®4% because the result of analysis was not adopted due to the data confliction.

## Declarations

### Author contribution statement

Mona Mohamad Mashal: Conceived and designed the experiments; Analyzed and interpreted the data; Contributed reagents, materials, analysis tools or data; Wrote the paper.

Basil Faisal Obeidat: Performed the experiments.

### Funding statement

This research did not receive any specific grant from funding agencies in the public, commercial, or not-for-profit sectors.

### Competing interest statement

The authors declare no conflict of interest.

### Additional information

No additional information is available for this paper.
